# Antibodies against *Borrelia burgdorferi* sensu lato among Adults, Germany, 2008–2011

**DOI:** 10.3201/eid2101.140009

**Published:** 2015-01

**Authors:** Hendrik Wilking, Volker Fingerle, Christiane Klier, Michael Thamm, Klaus Stark

**Affiliations:** Robert Koch-Institut, Berlin, Germany (H. Wilking, M. Thamm,. K. Stark);; German National Reference Centre for *Borrelia*, Oberschleißheim, Germany (V. Fingerle, C. Klier)

**Keywords:** Lyme borreliosis, Borrelia burgdorferi sensu lato, seroepidemiologic studies, adults, Germany, risk factors, seroprevalence, prevalence, tickborne disease, bacteria, Ixodes ricinus, Ixodes persulcatus

## Abstract

To assess *Borrelia burgdorferi* sensu lato (the cause of Lyme borreliosis) seropositivity in Germany, we tested serum samples from health survey (2008–2011) participants. Seroprevalence was 5.8% among women and 13.0% among men; infection risk was highest among persons >60 years of age. Public health interventions, including education about risk factors and preventive measures, are needed.

Lyme borreliosis, the most common tickborne disease in the Northern Hemisphere, is caused by infection with spirochetes of the *Borrelia* *burgdorferi* sensu lato (s.l.) complex. Five genospecies are known to be pathogenic for humans: *B. burgdorferi* sensu stricto (s.s.), *B. afzelii, B. garinii, B. bavariensis*, and *B. spielmanii* (*1*). In Europe, the bacterium is transmitted to humans through the bite of *Ixodes ricinus* ticks; in eastern Europe*, I. persulcatus* ticks can also transmit the bacterium.

In Europe, where the most common clinical manifestation of Lyme borreliosis is erythema migrans, followed by Lyme neuroborreliosis and Lyme arthritis (*2*), data are sparse regarding *B. burgdorferi* s.l. infection rates and risk factors (*3*). Persons of all ages are at risk for infection; however, surveillance data and prospective studies in Europe and the United States suggest that children and the elderly are particularly at risk (*4*–*6*). Population-based surveillance data suggest that Lyme borreliosis is endemic in eastern Germany: annual incidence is 20–35 cases/100,000 inhabitants (*7*). Regional differences in incidence are observed, but data cannot be easily compared because of reporting biases and differences in infection awareness.

The limited representativeness and comparability of Lyme borreliosis surveillance data are well documented (*8*). Under such conditions, population-based serosurveys with high representativeness can provide valid estimates of the force of infection (rate at which susceptible persons acquire Lyme borreliosis) and the lifetime risk for infection; however, seroprevalence estimates do not necessarily represent cases of clinical disease. In a population-based seroprevalence study among 1- to 17-year-old children in Germany, seroprevalence increased cumulatively by age (*9*). We present data on the prevalence and determinants of *B. burgdorferi* s.l. seropositivity among adults in Germany during 2008–2011.

## The Study

We estimated *B. burgdorferi* s.l. seroprevalence among participants of the German Health Interview and Examination Survey for Adults (DEGS). This nationwide cross-sectional survey assessed the health status of 18- to 79-year-old persons in Germany during 2008–2011 (*10*). The response rate was 48.4%; analysis of nonresponder questionnaires revealed high population representativeness. Data from standardized interviews were used to assess potential risk factors for seropositivity. Survey weights based on age, sex, residence in western or eastern Germany, and nationality (German vs. non-German) were calculated to correct for deviations from the German population statistics (December 31, 2010; http://www.destatis.de) and used throughout the analyses. The study was approved by the Ethical Review Board of the Medical School Charité, Berlin, Germany.

**Figure 1:**
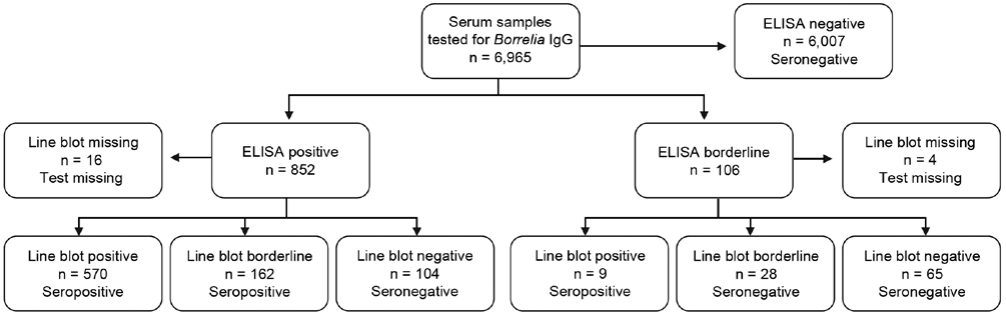
Categorization, according to ELISA and line blot test results, of serum samples tested for *Borrelia burgdorferi* sensu lato IgG, Germany, 2008–2011.

As recommended for serologic confirmation of clinical cases, serum samples were tested for the presence of *Borrelia* *burgdorferi* s.l. IgG. For screening, we used an ELISA based on *B. afzelii* extract antigen enriched with recombinant VlsE (an outer-surface protein) from *B. burgdorferi* s.s., *B. afzelii*, and *B. bavariensis*. ELISA-positive results were confirmed by line blot testing, which included purified antigens OspC, DbpA, and p83 from *B. afzelii*; recombinant VlsE from *B. burgdorferi* s.s. and *B. garinii*; and BmpA and DbpA from *B. garinii*, *B. bavariensis*, and *B. spielmanii*. Details regarding the tests are available in the Technical Appendix. All tests were performed/interpreted according to the manufacturer’s recommendations. To categorize samples by test results, we applied the rules shown in Figure 1.

We used sampling weights for all statistical analyses and accounted for the 2-stage sampling structure. Age-related prevalence was graphed and the values were smoothed by using the Lowess procedure of Stata 12.1 (StataCorp LP, College Station, TX, USA). We assessed differences between group prevalences (explanatory variables) by using the Wald test (univariable logistic regression) with 2-sided p values. Independent risk factors for seropositivity were investigated by using stepwise multivariable logistic regression. All plausible 2-way interactions were tested.

**Figure 2:**
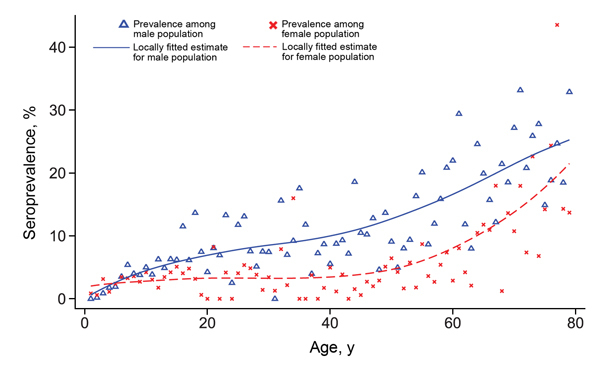
Estimated seroprevalence of *Borrelia burgdorferi* sensu lato IgG among the male and female population, Germany, 2008–2011. For comparison, results of Dehnert et al. (*9*), a previous study among children/adolescents <18 years of age, were added to the graph.

A total of 6,945 adults, representing 97.6% of the survey population with available blood samples, were included in the analysis. The overall weighted seroprevalence for *B. burgdorferi* s.l. was 9.4% (95% CI 8.4%–10.0%); seroprevalence was significantly higher among men (13.0%) than women (5.8%) (p<0.01). Seroprevalence among both sexes increased by age (Figure 2); the increase was low among 18- to 50-year-old participants, most pronounced among participants >59 years of age, and higher among women than men >59 years of age. Seropositivity reached 20.0% (95% CI 16.9%–23.6%) in 70- to 79-year-old participants.

Among participants >18 years of age, more than twice as many men than women were seropositive for *B. burgdorferi* s.l. (odds ratio 2.44, 95% CI 2.01–2.96) (Table). No significant interaction between sex and age was found (p = 0.075). Independent risk factors for seropositivity were residence in a rural area (p<0.001) and in southern Germany (p = 0.032). Non-German citizenship was negatively associated (p = 0.004) with seropositivity; having a dog/cat in the house was not associated with a higher risk for seropositivity. To facilitate comparison of our data with data from serosurveys lacking confirmatory testing, we have made our ELISA results available online (Technical Appendix Table).

**Table:** Stratified seroprevalence of *Borrelia burgdorferi* sensu lato IgG detected by combined ELISA and line blot testing in adults and results of weighted logistic regression analysis of potential risk factors for seropositivity, Germany, 2008–2011*

Characteristic†	No. positive/no. total†	Prevalence (95% CI)	Univariable analysis			Multivariable analysis	
			OR (95% CI)	p value		OR (95% CI)	p value
Sex							
F	240/3,614	5.8 (4.9–6.7)	Ref	Ref		Ref	Ref
M	501/3,331	13.0 (11.4–14.8)	2.44 (2.01–2.96)	<0.001		2.61 (2.15–3.16)	<0.001
Age group, y							
18–29	62/1,043	6.0 (4.5–8.0)	Ref	Ref		Ref	Ref
30–39	50/829	6.3 (4.4–9.0)	1.05 (0.64–1.69)	0.854		1.07 (0.67–1.72)	0.779
40–49	83/1,263	6.4 (5.0–8.2)	1.07 (0.72–1.58)	0.737		1.04 (0.69–1.55)	0.856
50–59	126/1,373	8.5 (6.8–10.7)	1.46 (1.01–2.10)	0.043		1.39 (0.97–1.99)	0.069
60–69	186/1,361	13.2 (10.9–15.9)	2.37 (1.65–3.40)	<0.001		2.37 (1.65–3.45)	<0.001
70–79	234/1,076	20.0 (16.9–23.6)	3.91 (2.77–5.51)	<0.001		4.02 (2.84–5.70)	<0.001
Residence location							
West‡	484/4.748	9.1 (8.0–10.4)	Ref	Ref		–	–
East§	257/2,197	10.4 (8.5–12.6)	1.15 (0.89–1.49)	0.273		–	–
North¶	181/1,767	9.0 (7.2–11.0)	1.11 (0.82–1.51)	0.479		1.16 (0.86–1.57)	0.318
Middle#	304/3,087	8.1 (6.7–9.8)	Ref	Ref		Ref	Ref
South**	256/2,091	11.2 (9.4–13.3)	1.43 (1.08–1.88)	0.011		1.34 (1.03–1.75)	0.032
Population of residence municipality							
<5,000	189/1,258	15.4 (12.8–18.4)	2.50 (1.85–3.30)	<0.001		2.13 (1.54–2.97)	<0.001
5,000 to <20,000	185/1,685	10.0 (8.0–12.5)	1.51 (1.11–2.07)	0.010		1.33 (0.96–1.84)	0.082
20,000 to <100,000	193/2,030	8.4 (6.9–10.2)	1.24 (0.92–1.66)	0.154		1.21 (0.88–1.67)	0.231
>100,000	174/1,972	6.9 (5.6–8.4)	Ref	Ref		Ref	Ref
Foreign national††							
No	721/6,528	10.0 (8.9–11.2)	Ref	Ref		Ref	Ref
Yes	18/396	4.3 (2.5–7.5)	0.41 (0.22–0.75)	0.004		0.54 (0.30–0.90)	0.041
Pet in household							
None	502/4,596	9.5 (8.4–10.7)	Ref	Ref		–	–
Any	217/2,182	9.3 (7.8–11.0)	0.98 (0.80–1.20)	0.834		–	–
Dog							
No	639/5,909	9.4 (8.4–10.6)	Ref	Ref		–	–
Yes	80/858	9.3 (7.0–12.2)	0.98 (0.71–1.35)	0.909		–	–
Cat							
No	622/5,886	9.2 (8.2–10.3)	Ref	Ref		–	–
Yes	119/1,077	10.3 (8.1–13.0)	1.13 (0.87–1.47)	0.356		–	–
Other animals							
No	655/ 6,001	9.7 (8.8–10.6)	Ref	Ref		–	–
Yes	64/766	7.7 (5.8–10.1)	0.78 (0.56–1.07)	0.127		–	–
Total	741/6,945	9.4 (8.4–10.0)	–	–		–	–

*OR, odds ratio; Ref, reference; –, not included in the final model. †Unweighted. ‡Western states: Baden-Württemberg, Bavaria, Bremen, Hamburg, Hesse, Lower Saxony, Northrhine-Westfalia, Rhineland-Palatinate, Saarland, Schleswig-Holstein. §Eastern states: Berlin, Brandenburg, Mecklenburg–West Pomerania, Saxony, Saxony-Anhalt, Thuringia. ¶Northern states: Schleswig-Holstein, Hamburg, Lower Saxony, Bremen, Berlin, Brandenburg, Mecklenburg-West Pomerania. #Middle states: Nordrhine-Westfalia, Hesse, Saxony, Saxony-Anhalt, Thuringia. **Southern states: Rhineland-Palatinate, Baden-Württemberg, Bavaria, Saarland. ††Defined as persons holding a foreign citizenship.

## Conclusions

*B. burgdorferi* s.l. infections are common in Germany; Lyme borreliosis is endemic in all regions, but case numbers are highest in southern Germany. Previously identified risk factors for *B. burgdorferi* s.l. seropositivity in children (male sex and living in rural areas, small-sized towns, or southern Germany) were identified as risk factors for seropositivity among adults in our study. Holding a cat was previously shown to be a risk factor for children/adolescents (*9*), but was not a risk factor in our study. Seroprevalence among the oldest age group indicates that at least one fifth of the German population becomes infected with *B. burgdorferi* s.l. during their lifetime.

*B. burgdorferi* s.l. IgG seroprevalence among blood donors in Italy (4.9%; n = 365) (*11*) and Romania (4.3%; n =1,598) (*12*) was lower than the seroprevalence in our study. Prevalences higher than those in our study have been shown in serosurveys in areas of high disease endemicity in southwestern Germany (16.9%; n = 1,228) (*13*) and Finland (19.3%; n = 3,248) (*14*). In serosurveys of persons with high exposure to ticks (e.g., forestry and agricultural workers), similar or higher seroprevalence rates have been described.

Seroprevalence rates among men in our study were strikingly higher than rates among women, indicating that tick contact/spirochete transmission is more frequent among men. Prospective studies in Germany and Sweden and surveillance data from Germany show no differences in clinical cases (except only a slight preponderance among women) that would point to substantial sex-specific differences in the development of clinical disease (*5*–*7*).

The age distribution for seropositivity reflects the population’s cumulative exposure to *B. burgdorferi* s.l.. An increased risk for infection among children and persons >59 years of age suggests that leisure activities/behaviors rather than occupational exposure are the main risk factor for infection. Alternatively, these findings might be explained by a birth-cohort effect, in which the force of infection was lower during 1950–1990.

Persons living in urbanized areas had a lower probability for *B. burgdorferi* s.l. seropositivity, suggesting that exposure to infected ticks is higher in rural areas. However, urban populations are also at substantial risk for infection. Seropositivity is not equivalent to clinical disease; thus, seropositivity rates among the different population groups may not necessarily reflect the true effect of infection on disease burden. Furthermore, a US study showed that persons can be consecutively infected by different *B. burgdorferi* strains and experience clinical manifestations with each infection (*15*).

Our seroprevalence estimates can be used, within the context of clinical diagnoses, to assess the likelihood of Lyme borreliosis in persons with test results positive for *B. burgdorferi* s.l. IgG. To reduce the incidence and disease burden of Lyme borreliosis, enhanced public health interventions are needed, including education campaigns targeted to parents, children, and the elderly about potential risk factors and preventive measures for Lyme borreliosis.

Technical Appendix Stratified seroprevalence of *Borrelia burgdorferi* sensu lato IgG detected by ELISA in adults and results of weighted bivariate logistic regression analysis of potential risk factors for seropositivity, Germany, 2008–2011.

## References

[1] Fingerle V, Schulte-Spechtel UC, Ruzic-Sabljic E, Leonhard S, Hofmann H, Weber K, Epidemiological aspects and molecular characterization of *Borrelia burgdorferi* s.l. from southern Germany with special respect to the new species *Borrelia spielmanii* sp. nov. Int J Med Microbiol. 2008;298:279–90. 10.1016/j.ijmm.2007.05.002 17616434

[2] Strle F, Stanek G. Clinical manifestations and diagnosis of Lyme borreliosis. Curr Probl Dermatol. 2009;37:51–110. 19367097 10.1159/000213070

[3] Rizzoli A, Hauffe H, Carpi G, Vourc HG, Neteler M, Rosa R. Lyme borreliosis in Europe. Euro Surveill. 2011;16:19906 . 21794218

[4] Bacon RM, Kugeler KJ, Mead PS. Surveillance for Lyme disease—United States, 1992–2006. MMWR Surveill Summ. 2008;57:1–9. 18830214

[5] Berglund J, Eitrem R, Ornstein K, Lindberg A, Ringer A, Elmrud H, An epidemiologic study of Lyme disease in southern Sweden. N Engl J Med. 1995;333:1319–24. 10.1056/NEJM199511163332004 7566023

[6] Huppertz HI, Böhme M, Standaert SM, Karch H, Plotkin SA. Incidence of Lyme borreliosis in the Würzburg region of Germany. Eur J Clin Microbiol Infect Dis. 1999;18:697–703. 10.1007/s100960050381 10584895

[7] Wilking H, Stark K. Trends in surveillance data of human Lyme borreliosis from six federal states in eastern Germany, 2009–2012. Ticks Tick Borne Dis. 2014;5:219–24. 10.1016/j.ttbdis.2013.10.010 24656810

[8] Ertel SH, Nelson RS, Cartter ML. Effect of surveillance method on reported characteristics of Lyme disease, Connecticut, 1996–2007. Emerg Infect Dis. 2012;18:242–7. 10.3201/eid1802.101219 22304873 PMC3310440

[9] Dehnert M, Fingerle V, Klier C, Talaska T, Schlaud M, Krause G, Seropositivity of Lyme borreliosis and associated risk factors: a population-based study in children and adolescents in Germany (KiGGS). PLoS ONE. 2012;7:e41321. 10.1371/journal.pone.0041321 22905101 PMC3419690

[10] Scheidt-Nave C, Kamtsiuris P, Gosswald A, Holling H, Lange M, Busch MA, German health interview and examination survey for adults (DEGS)—design, objectives and implementation of the first data collection wave. BMC Public Health. 2012;12:730. 10.1186/1471-2458-12-730 22938722 PMC3490742

[11] Tomao P, Ciceroni L, D'Ovidio MC, De RM, Vonesch N, Iavicoli S, Prevalence and incidence of antibodies to *Borrelia burgdorferi* and to tick-borne encephalitis virus in agricultural and forestry workers from Tuscany, Italy. Eur J Clin Microbiol Infect Dis. 2005;24:457–63. 10.1007/s10096-005-1348-0 15948001

[12] Hristea A, Hristescu S, Ciufecu C, Vasile A. Seroprevalence of *Borrelia burgdorferi* in Romania. Eur J Epidemiol. 2001;17:891–6. 10.1023/A:1015600729900 12081110

[13] Hassler D, Zoller L, Haude M, Hufnagel HD, Sonntag HG. Lyme borreliosis in an endemic region in Europe: prevalence of antibodies and clinical spectrum [in German]. Dtsch Med Wochenschr. 1992;117:767–74 . 10.1055/s-2008-1062374 1587208

[14] Carlsson SA, Granlund H, Nyman D, Wahlberg P. IgG seroprevalence of Lyme borreliosis in the population of the Aland Islands in Finland. Scand J Infect Dis. 1998;30:501–3 and. 10.1080/00365549850161520 10066053

[15] Nadelman RB, Hanincova K, Mukherjee P, Liveris D, Nowakowski J, McKenna D, Differentiation of reinfection from relapse in recurrent Lyme disease. N Engl J Med. 2012;367:1883–90 . 10.1056/NEJMoa1114362 23150958 PMC3526003

