# Supplementary material for: Antibodies against Borrelia burgdorferi sensu lato among Adults, Germany, 2008–2011
Source: Emerg Infect Dis. 2015 Jan;21(1):107–10. doi: 10.3201/eid2101.140009 (PMC4285254; doi:10.3201/eid2101.140009)
Supplement: Technical Appendix — Stratified seroprevalence of Borrelia burgdorferi sensu lato IgG detected by ELISA in adults and results of weighted bivariate logistic regression analysis of potential risk factors for seropositivity, Germany, 2008–2011. [file 14-0009-Techapp-s1.pdf]

Article DOI: http://dx.doi.org/10.3201/eid2101.140009

Antibodies against *Borrelia burgdorferi* sensu lato among Adults, Germany, 2008–2011

Technical Appendix

The serum samples were tested at the National Reference Centre for *Borrelia* for the presence of anti–*Borrelia burgdorferi* sensu lato IgG antibodies. As an initial test an ELISA (Enzygnost Lyme link VlsE/IgG; Siemens Healthcare Diagnostics GmbH, Eschborn, Germany) was applied. It is based on a detergent extract from cultured *B. afzelii* (strain PKo) mixed with recombinant VlsE from *B. burgdorferi* sensu stricto (strain B31), *B. afzelii* (strain PKo), and *B. bavariensis* (strain PBi). The test was automatically processed on a BEP III (Siemens Healthcare Diagnostics) and interpreted as recommended by the manufacturer. Validation studies for this ELISA have been published (*1*,*2*).

For confirmation of ELISA test–positive and borderline tests samples, a line blot was performed (Borrelia Europe plus TpN17 LINE IgG; Virotech, Rüsselsheim, Germany). It includes the purified antigens OspC, DbpA, and p83 (all from *B. afzelii* strain PKo) and additionally recombinant antigens VlsE (from *B. burgdorferi* sensu stricto strain B31 and *B. garinii* strain IP90), BmpA (PKo), DbpA (from *B. garinii* strain PBr, *B. bavariensis* strain PBi, and *B. spielmanii*). Antigens are bound separately to a nitrocellulose membrane either as single antigens or, in the case of VlsE and DbpA, as a mix of the respective antigens. The test was performed and interpreted according to the manufacturer's recommendations.

References

1. Ang CW, Notermans DW, Hommes M, Simoons-Smit AM, Herremans T. Large differences between test strategies for the detection of anti-*Borrelia* antibodies are revealed by comparing eight ELISAs and five immunoblots. Eur J Clin Microbiol Infect Dis. 2011;30:1027–32. PubMed http://dx.doi.org/10.1007/s10096-011-1157-6

2. Marangoni A, Moroni A, Accardo S, Cevenini R. *Borrelia burgdorferi* VlsE antigen for the serological diagnosis of Lyme borreliosis. Eur J Clin Microbiol Infect Dis. 2008;27:349–54. PubMed http://dx.doi.org/10.1007/s10096-007-0445-7

Technical Appendix Table. Stratified seroprevalence of *Borrelia burgdorferi* sensu lato IgG detected by ELISA (initial test only) in adults 18- to 79–years if age and results of weighted bivariate logistic regression analysis of potential risk factors for seropositivity, Germany, 2008–2011*

Characteristics†	No. positive/no. total†	Prevalence (95% CI)	Univariable analysis OR (95% CI)	Univariable analysis p value	Multivariable analysis OR (95% CI)	Multivariable analysis p value
Sex						
F	295/3,619	7.1 (6.2–8.2)	Ref	Ref	Ref	Ref
M	557/3,346	14.5 (12.8–16.4)	2.21 (1.84 – 2.66)	<0.001	2.34 (1.95–2.82)	<0.001
Age group, y						
18–29	68/1,044	6.9 (5.3–8.9)	Ref	Ref	Ref	Ref
30–39	66/831	8.2 (6.1–11.1)	1.22 (0.79–1.87)	0.374	1.25 (0.82–1.91)	0.303
40–49	98/1,270	8.0 (6.4–10.0)	1.18 (0.83–1.67)	0.351	1.15 (0.80–1.64)	0.454
50–59	149/1,375	9.8 (7.8–12.1)	1.46 (1.04–2.06)	0.029	1.40 (1.01–1.95)	0.046
60–69	209/1,364	14.4 (12.0–17.2)	2.28 (1.64–3.16)	<0.001	2.28 (1.64–3.16)	<0.001
70–79	262/1,081	21.9 (18.6–25.7)	3.80 (2.76–5.23)	<0.001	3.84 (2.77–5.32)	<0.001
Residence location in Germany						
West‡	569/4,761	10.5 (9.3–11.9)	Ref	Ref	–	–
East§	283/2,204	11.8 (9.8–14.2)	1.14 (0.89–1.46)	0.273	–	–
North¶	200/1,771	10.1 (8.3–12.2)	1.05 (0.78–1.39)	0.761	1.08 (0.81–1.44	0.587
Middle#	358/3,094	9.7 (8.1–11.5)	Ref	Ref	Ref	Ref
South**	294/2,100	12.7 (10.9–14.9)	1.37 (1.05–1.77)	0.020	1.30 (1.00–1.69)	0.049
Population of residence municipality						
<5,000	207/1,264	16.8 (13.9–20.3)	2.23 (1.69–2.95)	<0.001	1.88 (1.37–2.60)	<0.001
5,000 to <20,000	205/1,687	11.2 (9.0–13.8)	1.38 (1.03–1.86)	<0.001	1.21 (0.89–1.64)	0.220
20,000 to <100,000	237/2,037	10.0 (8.3–12.1)	1.23 (0.94–1.61)	0.154	1.19 (0.89–1.58)	0.236
>100,000	203/1,977	8.3 (7.0–9.8)	Ref	Ref	Ref	Ref
Foreign national††						
No	829/6,547	11.6(10.4–12.8)	Ref	Ref	Ref	Ref
Yes	21/397	4.5 (2.6–7.7)	0.36 (0.20–0.64)	0.001	0.45 (0.25–0.80)	0.007
Pet in household						
No pet	589/4,613	11.0 (9.9–12.2)	Ref	Ref	–	–
Any pet	240/2,185	10.6 (9.0–12.5)	0.96 (0.80–1.16)	0.676	–	–
Dog						
No	741/5,928	11.0 (9.9–12.2)	Ref	Ref	–	–
Yes	88/859	10.2 (7.8–13.1)	0.92 (0.68–1.23)	0.562	–	–
Cat						
No	698/5,708	10.7 (9.6–11.9)	Ref	Ref	–	–
Yes	131/1,079	11.8 (9.4–14.8)	1.12 (0.86–1.45)	0.394	–	–
Other animals						
No	758/6,020	11.2 (10.0–12.4)	Ref	Ref	–	–
Yes	71/767	8.8 (6.7–11.5)	0.77 (0.57–1.04)	0.086	–	–
Total	852/6,965	11.0 (9.7–12.0)	–	–	–	–

*OD, odds ratio; Ref, reference, –,not included in the final mode..
†Unweighted.
‡Western states: Baden-Württemberg, Bavaria, Bremen, Hamburg, Hesse, Lower Saxony, Northrhine-Westfalia, Rhineland-Palatinate, Saarland, Schleswig-Holstein.
§Eastern states: Berlin, Brandenburg, Mecklenburg-West Pomerania, Saxony, Saxony-Anhalt, Thuringia.
¶Northern states: Schleswig-Holstein, Hamburg, Lower Saxony, Bremen, Berlin, Brandenburg, Mecklenburg-West Pomerania.
#Middle states: Nordrhine-Westfalia, Hesse, Saxony, Saxony-Anhalt, Thuringia.
**Southern states: Rhineland-Palatinate, Baden-Württemberg, Bavaria, Saarland.
††Defined as persons holding a foreign citizenship.
